# The relationship between dietary sodium intake and all-cause mortality in patients with non-alcoholic fatty liver disease: a cohort study from NHANES 2003–2018

**DOI:** 10.3389/fnut.2025.1530025

**Published:** 2025-04-23

**Authors:** Jiajun Li, Sile Wan, Xianyu Dai, Yifeng Cui, Zhaoyang Lu

**Affiliations:** ^1^Department of Hepatic Surgery, The First Affiliated Hospital of Harbin Medical University, Harbin, China; ^2^Urology Department, First Hospital of Jilin University, Changchun, China

**Keywords:** sodium intake, non-alcoholic fatty liver disease, National Health and Nutrition Examination Survey, all-cause mortality, dose-response relationship

## Abstract

**Background:**

The relationship between sodium intake and the incidence and mortality of non-alcoholic fatty liver disease (NAFLD) is underexplored in nutritional epidemiology, highlighting the need for further research.

**Methods:**

This longitudinal cohort study analyzed data from 13,853 Participants aged 20 and older from the National Health and Nutrition Examination Survey (NHANES) (2003–2018), including 4,465 participants with NAFLD. We collected comprehensive data on mortality, dietary sodium intake, and relevant covariates. Logistic regression assessed the relationship between sodium consumption and NAFLD incidence, while Cox regression and smooth curve fitting explored sodium intake’s link to all-cause mortality among Participants with NAFLD.

**Results:**

After adjusting for confounders, logistic regression revealed a positive association between higher sodium intake and NAFLD incidence (OR = 1.16, 95% CI = 1.11, 1.21). Adjusted odds ratios for the second (Q2), third (Q3), and fourth (Q4) quartiles of sodium intake were 0.91, 1.23, and 1.52, respectively. Smooth curve fitting and threshold analysis revealed a non-linear association between sodium intake and NAFLD risk, with an inflection point at 2.49 g/d, above which NAFLD risk significantly increased. In Cox regression, sodium intake was inversely correlated with all-cause mortality in Participants with NAFLD (HR = 0.87, 95% CI = 0.80, 0.96), with adjusted hazard ratios for Q2, Q3, and Q4 being 0.79, 0.66, and 0.63, respectively. A nonlinear model indicated a threshold effect, revealing a correlation between dietary sodium intake and mortality risk (*p* = 0.001). We identified a threshold intake of 3.5 grams per day (equivalent to 8.9 grams of sodium chloride): below this, each unit increase in sodium intake was associated with a 16% reduction in mortality risk (HR = 0.84, 95% CI = 0.80, 0.90). For intakes above this threshold, no significant relationship with mortality risk was observed (HR = 0.99, 95% CI = 0.90, 1.08).

**Conclusion:**

This study suggests that higher sodium intake in individuals with NAFLD is associated with increased disease incidence but decreased all-cause mortality. The dose–response relationship between sodium intake and mortality risk exhibited a nonlinear pattern, with a critical inflection point around 3.5 grams per day.

## Introduction

Non-alcoholic fatty liver disease (NAFLD) is a metabolic disorder defined by the abnormal accumulation of fat within the liver (1). It has emerged as a primary contributor to chronic liver disease globally, with a prevalence rate reaching 25% worldwide (2). In the United States, more than 30% of the population is affected by NAFLD (3). Currently, non-alcoholic steatohepatitis (NASH), a progression of NAFLD, has emerged as the fastest-growing cause of hepatocellular carcinoma globally (4, 5), while NAFLD-related liver failure has become the second most common reason for liver transplantation in developed nations (6). Moreover, NAFLD is closely linked to negative outcomes of chronic conditions like liver disease, cardiovascular disease, and diabetes, creating a substantial global health and economic cost (7). The etiology of NAFLD is quite intricate, with metabolic disorders such as obesity, hypertension, and dyslipidemia being major contributing factors (8). Growing evidence indicates that dietary consumption plays a vital role in the onset of NAFLD (9, 10).

Over the past few decades, dietary sodium intake has been acknowledged as a factor associated with cardiovascular diseases and increased risk of early mortality (11). Excessive sodium intake is believed to shorten lifespan, with approximately 1.8 million global deaths in 2019 attributed to sodium consumption (9). So far, research investigating the link between sodium intake and the incidence of NAFLD remains limited. We reviewed articles from the past 15 years and identified nine relevant studies (Supplementary Table S1), but the conclusions of these studies remain controversial. Currently, there is a deficiency of research investigating the association between sodium intake and all-cause mortality risk in patients with NAFLD, leaving the nature of this association unclear. This study intends to systematically explore the relationship between sodium intake and the incidence and all-cause mortality of NAFLD in American adults by comprehensively reviewing the literature and analyzing data from The National Health and Nutrition Examination Survey (NHANES), thus addressing this gap in the literature.

## Methods

### Study design and participants

**Figure 1:**
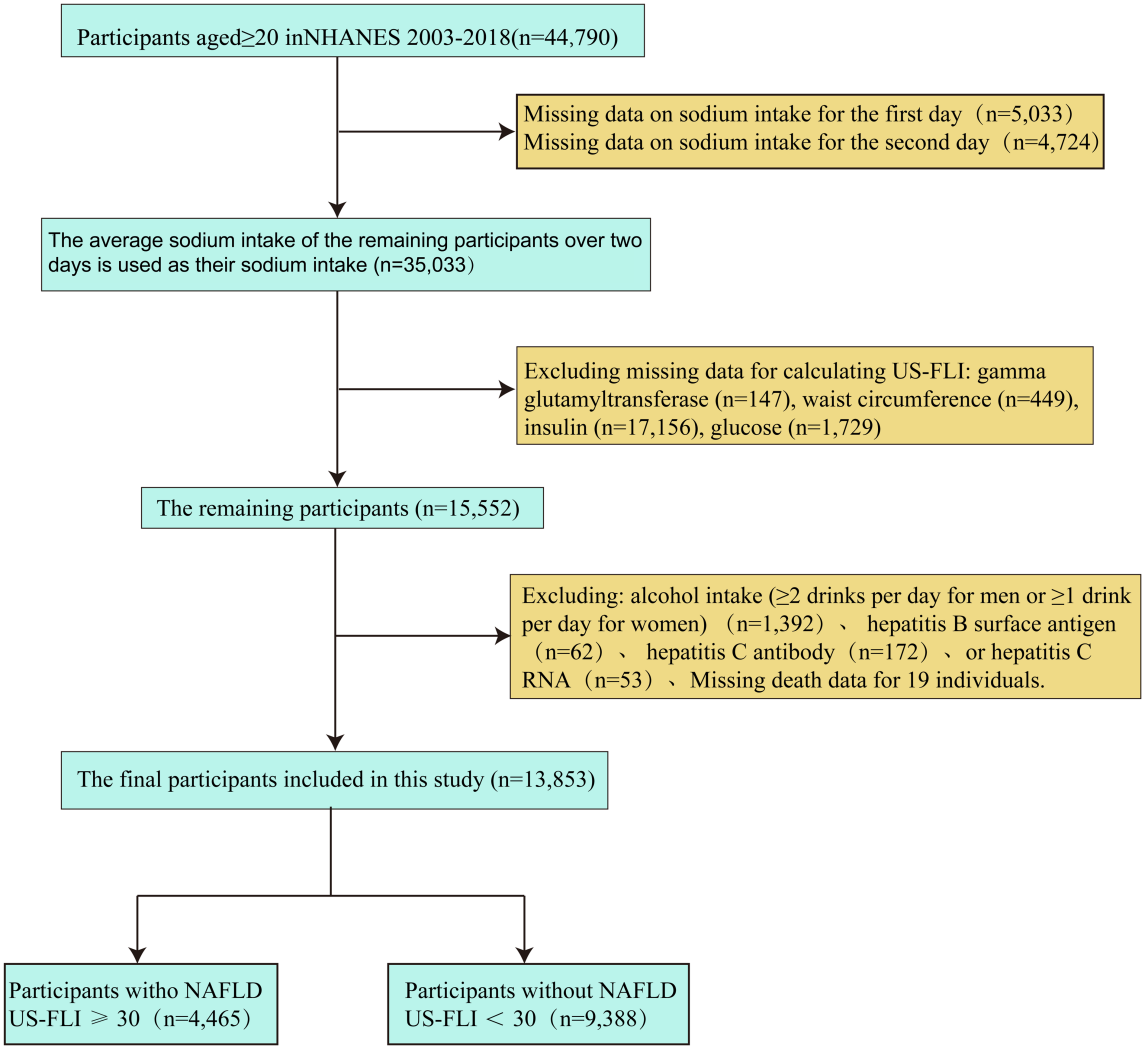
Flow chart illustrating selection of the study population in NHANES from 2003 to 2018.

NHANES is a comprehensive program conducted by the National Center for Health Statistics (NCHS), utilizing a sophisticated multi-tiered probability sampling design to gather data on the non-institutionalized population of the country biennially. Our prospective cohort study utilized NHANES data from 2003 to 2018 and adhered to the ethical standards set forth by the CDC Institutional Review Board, ensuring the rights and confidentiality of participants. All participants gave informed consent before participation, confirming their voluntary involvement and understanding of the study’s objectives and procedures. To guarantee the precision and dependability of the study population, strict exclusion criteria were applied, eliminating individuals with incomplete sodium intake records, missing mortality information, or absent data for NAFLD diagnostic indices. The methods for participant selection, including inclusion and exclusion criteria, are detailed in the flowchart (Figure 1).

### Measurement of dietary sodium intake

In our study, dietary sodium intake was obtained through dietary interviews conducted at the Mobile Examination Center (MEC). All qualifying NHANES participants completed two 24-h dietary recall interviews, detailing the types and amounts of food consumed in the 24 h preceding the interview. The accuracy of this method has been validated. During the interviews, participants provided their sodium intake, and the nutritional composition of all foods was calculated using the USDA Food and Nutrition Database System (FNDDS) to ensure standardized nutritional assessment. In this research, the daily sodium intake for each participant was assessed by averaging the two dietary recall interviews.

### Definition of NAFLD

NAFLD is determined using the formula for the United States Fatty Liver Index (US-FLI) = e^y^/(1 + e^y^) × 100, y = −0.8073 × non-Hispanic Black + 0.3458 × Mexican American + 0.0093 × age + 0.6151 × log_e_ (gamma-glutamyl transferase) + 0.0249 × waist circumference + 1.1792 × log_e_ (insulin) + 0.8242 × log_e_ (glucose) − 14.7812. The variables for ‘non-Hispanic Black’ and ‘Mexican American’ were coded as 1 if the participant identified with that ethnicity and 0 otherwise (12). Fatty liver is defined by a US-FLI score of ≥30, as recommended (13, 14). NAFLD is identified by a US-FLI score of ≥30, discounting other established reasons for chronic liver disease. These include viral hepatitis, indicated by positive markers such as hepatitis B surface antigen, hepatitis C antibody, or hepatitis C RNA, and substantial alcohol consumption (≥2 drinks per day for men or ≥1 drink per day for women).

### Assessment of covariates

In this study, participants were stratified into three age groups: 20–39 years, 40–59 years, and 60 years or older. Racial and ethnic classifications encompassed non-Hispanic White, non-Hispanic Black, Mexican American, and other groups. Education levels were categorized as less than high school, high school or equivalent, and college or higher. The poverty income ratio (PIR) was divided into three categories: less than 1.0, 1.0 to 3.0, and greater than 3.0. Smoking status was classified into three groups: never smokers (individuals who have smoked fewer than 100 cigarettes in their lifetime), current smokers (those who have smoked more than 100 cigarettes), and former smokers (individuals who have smoked over 100 cigarettes and are currently abstinent). Activity levels were evaluated based on reported moderate work activity from the survey questionnaire, classified as “yes” or “no.” Body mass index (BMI) was categorized as less than 25.0, between 25 and 30, and 30 or greater. Total energy intake was derived from participants’ 24-h dietary recall interviews. Medical histories of hypertension, high cholesterol, and diabetes were collected through self-reports. Laboratory indicators included alanine aminotransferase (ALT), aspartate aminotransferase (AST), total cholesterol (TC), triglycerides (TG), low-density lipoprotein cholesterol (LDL), high-density lipoprotein cholesterol (HDL), and gamma-glutamyl transferase (GGT).

### Statistical analysis

In the present research, all analyses considered the intricate sampling design of NHANES and included sample weights to ensure a precise reflection of the population. Survival time was calculated from baseline until death, loss to follow-up, or December 31, 2019, measured in months. Continuous variables were expressed as means weighted by the survey, accompanied by their respective 95% confidence intervals (CIs), while categorical variables were presented as percentages weighted by the survey, along with the corresponding 95% CIs. We employed survey-weighted linear regression to assess differences in continuous variables between groups and used survey-weighted chi-square tests to analyze differences in categorical variables. For dietary sodium intake, we selected the lowest 25% as a reference to explore its transparency and impact on risk assessment. Survey-weighted multivariable logistic regression was used to analyze the relationship between sodium intake and the incidence of NAFLD, with results presented as odds ratios (ORs) and 95% CIs. Additionally, survey-weighted multivariable Cox regression was employed to assess the relationship between sodium intake and all-cause mortality in NAFLD participants, with results reported as hazard ratios (HRs) and 95% CIs. Survival differences between sodium intake quartiles were illustrated using Kaplan–Meier curves (log-rank test).

To control for confounding factors, we utilized three regression models: (1) unadjusted model; (2) adjusted for gender, age, race, education level, and poverty income ratio (PIR); (3) further adjusted for physical activity, total energy intake, smoking, BMI, hypertension, diabetes, high cholesterol, ALT, AST, TC, TG, LDL, HDL, and GGT.

Moreover, we conducted multivariable-adjusted subgroup analyses based on factors such as age, gender, race/ethnicity, BMI, hypertension, high cholesterol, and diabetes, using the *p*-values of interaction terms between sodium intake and stratifying factors to estimate the significance of interactions. We also employed multivariable-adjusted smooth curve fitting based on generalized additive models and restricted cubic spline Cox models to further assess possible non-linear associations between sodium intake and mortality (15–17). If a non-linear relationship was detected, recursive algorithms (18, 19) would be used to calculate inflection points, and adjusted two-segment Cox proportional hazards models (20–22) would be analyzed on either side of the inflection point. All statistical analyses were performed using R version 4.3.1 and EmpowerStats, with statistical significance established at a two- sided *p*-value of less than 0.05.

## Results

### Selection of study population

Among the 80,312 NHANES participants from 2003 to 2018, there were 44,790 participants aged over 20 years. Following the elimination of participants with incomplete data on sodium intake and the necessary calculations for US-FLI, 15,552 participants remained. Further exclusions were made for heavy drinkers and individuals with hepatitis B or hepatitis C. As a result, a final total of 13,853 participants was included, comprising 4,465 NAFLD participants and 9,388 non-NAFLD participants.

### Baseline characteristics

Table 1 presents a summary of the baseline characteristics of the 13,853 participants, stratified by their NAFLD status. Among them, 4,465 individuals (32.2%) had NAFLD. We observed that participants with NAFLD were more likely to be male, predominantly non-Hispanic White, and primarily aged between 40 and 59 years. This population had a higher prevalence of obesity (BMI ≥ 30 kg/m^2^), typically attained a college education or above, and had better economic status (PIR > 3).

**Table 1:** Characteristics of the study population (*n* = 13,853).

Characteristic	Overall *N* = 13,853	NAFLD *N* = 4,465	Non-NAFLD *N* = 9,388	*p* value
Age group (%)				<0.001
20–39	4,514 (32.58)	994 (22.27)	3,474 (37.01)	
40–59	5,335 (38.51)	1813 (40.62)	3,531 (37.61)	
>59	4,004 (28.91)	1,658 (37.11)	2,383 (25.38)	
Gender, *n* (%)				<0.001
Male	6,191 (44.69)	2,377 (53.23)	3,851 (41.02)	
Female	7,662 (55.31)	2088 (46.77)	5,537 (58.98)	
Race/ethnicity, *n* (%)				<0.001
Non-Hispanic White	9,815 (70.86)	3,139 (70.29)	6,674 (71.09)	
Non-Hispanic Black	1,402 (10.12)	514 (11.52)	894 (9.52)	
Hispanic	993 (7.16)	354 (7.92)	642 (6.84)	
Other	1,643 (11.86)	458 (10.27)	1,178 (12.55)	
Education levels, *n* (%)				<0.001
Less than High school	2010 (14.51)	790 (17.67)	1,234 (13.14)	
High school or equivalent	3,113 (22.47)	1,079 (24.17)	2040 (21.73)	
College or above	8,722 (63.02)	2,596 (58.16)	6,114 (65.13)	
Family income–poverty ratio, *n* (%)				<0.001
<1.0	1,627 (11.75)	566 (12.68)	1,065 (11.35)	
1.0–3.0	4,974 (35.91)	1733 (38.79)	3,255 (34.67)	
>3.0	7,252 (52.34)	2,166 (48.53)	5,068 (53.98)	
Smoking status, *n* (%)				0.226
Current smokers	2,283 (16.48)	693 (15.52)	1,585 (16.89)	
Former smokers	3,778 (27.27)	1,210 (27.11)	2,566 (27.34)	
Never smokers	7,792 (56.25)	2,562 (57.37)	5,237 (55.77)	
Moderate work activity				0.248
Yes	4,936 (35.64)	1,632 (36.55)	3,309 (35.25)	
No	8,917 (64.36)	2,833 (63.45)	6,079 (64.75)	
BMI, kg/m^2^	29.15 ± 6.75	29.429 ± 6.96	28.559 ± 6.23	<0.001
BMI (%)				<0.001
< 25	2,634 (19.02)	905 (20.26)	1735 (18.48)	
25–30	4,119 (29.74)	1,443 (32.32)	2,689 (28.62)	
≥ 30	7,100 (51.24)	2,117 (47.42)	4,964 (52.90)	
Total energy, kcal	1985.46 ± 780.54	1994.28 ± 796.16	1981.35 ± 772.99	0.961
Hypertension, *n* (%)				0.697
Yes	5,649 (40.78)	1806 (40.45)	3,842 (40.93)	
No	8,204 (59.22)	2,659 (59.55)	5,546 (59.07)	
High cholesterol, *n* (%)				0.268
Yes	5,500 (39.70)	1729 (38.73)	3,766 (40.12)	
No	8,353 (60.30)	2,736 (61.27)	5,622 (59.88)	
Diabetes, *n* (%)				0.831
Yes	2,104 (15.18)	671 (15.04)	1,431 (15.24)	
No	11,749 (84.82)	3,794 (84.96)	7,957 (84.76)	
Sodium Intake, g	3.29 ± 1.43	3.37 ± 1.49	3.25 ± 1.41	<0.001
ALT, U/L	23.93 ± 17.10	23.92 ± 21.01	23.93 ± 14.87	0.981
AST, U/L	24.54 ± 18.68	24.62 ± 22.97	24.51 ± 16.25	0.704
TC, mg/dL	194.07 ± 42.58	193.51 ± 41.91	194.34 ± 42.89	0.732
TG, mg/dL	128.71 ± 108.54	125.71 ± 102.06	130.13 ± 111.49	0.383
LDL, mg/dL	114.16 ± 36.40	113.69 ± 36.15	114.38 ± 36.53	0.676
HDL, mg/dL	48.28 ± 22.71	48.65 ± 23.19	48.11 ± 22.49	0.917
GGT, U/L	27.42 ± 38.39	26.71 ± 38.08	27.76 ± 38.53	0.428

NAFLD, nonalcoholic fatty liver disease; NHANES, National Health and Nutrition Examination Survey; BMI, body mass index; ALT, alanine aminotransferase; AST, asparate aminotransferase; TC, total cholesterol; TG, triglyceride; LDL, low-density lipoprotein cholesterol; HDL, high-density lipoprotein cholesterol; GGT, Gamma Glutamyl Transferase.

### Relationship between sodium consumption and NAFLD

The results indicated a significant positive correlation between sodium intake and the occurrence of NAFLD. After adjusting for all covariates (Model 3), ORs and 95% CIs for NAFLD across different sodium intake quartiles (Q1-Q4) were as follows: 1.00 (reference group), 0.91 (0.79, 1.06), 1.23 (1.06, 1.44), and 1.52 (1.30, 1.79), with a trend test *p*-value of <0.001 (Table 2). When sodium intake was analyzed as a continuous variable, the association remained significant, with each unit increase in sodium intake correlating with a 16% increase in the prevalence of NAFLD (OR: 1.16, 95% CI: 1.11, 1.21) (Table 2).

**Table 2:** ORs (95% CIs) for NAFLD based on sodium intake, weighted analysis.

Sodium intake	Model 1			Model 2			Model 3		
	OR	95% CI	*p*-value	OR	95% CI	*p*-value	OR	95% CI	*p*-value
Sodium intake^a^	1.13	1.10, 1.16	<0.001	1.16	1.11, 1.21	<0.001	1.16	1.11, 1.21	<0.001
Sodium intake^b^									
Q1	1.00	-	–	1.00	–	-	1.00	–	–
Q2	0.85	0.74, 0.98	0.027	0.92	0.79, 1.06	0.254	0.91	0.79, 1.06	0.249
Q3	1.11	0.96, 1.28	0.170	1.24	1.06, 1.45	0.008	1.23	1.06, 1.44	0.009
Q4	1.36	1.20, 1.54	<0.001	1.53	1.31, 1.79	<0.001	1.52	1.30, 1.79	<0.001
P for trend			<0.001			<0.001			<0.001

OR, odds ratio; CI, confidence interval. Model 1: Non-adjusted. Model 2: Adjusted for sex, age, race, education, PIR. Model 3: Adjusted for sex, age, race, education, PIR, BMI, physical activity, smoking, total caloric intake, hypertension, High cholesterol, diabetes, ALT, AST, TC, TG, LDL, HDL, GGT.

a Each 1 Each 1 unit increase in sodium intake.

b Q1: <2.3 g/d; Q2: 2.3–3.1 g/d; Q3: 3.1–4.0 g/d; Q4: ≥4.0 g/d.

**Figure 2:**
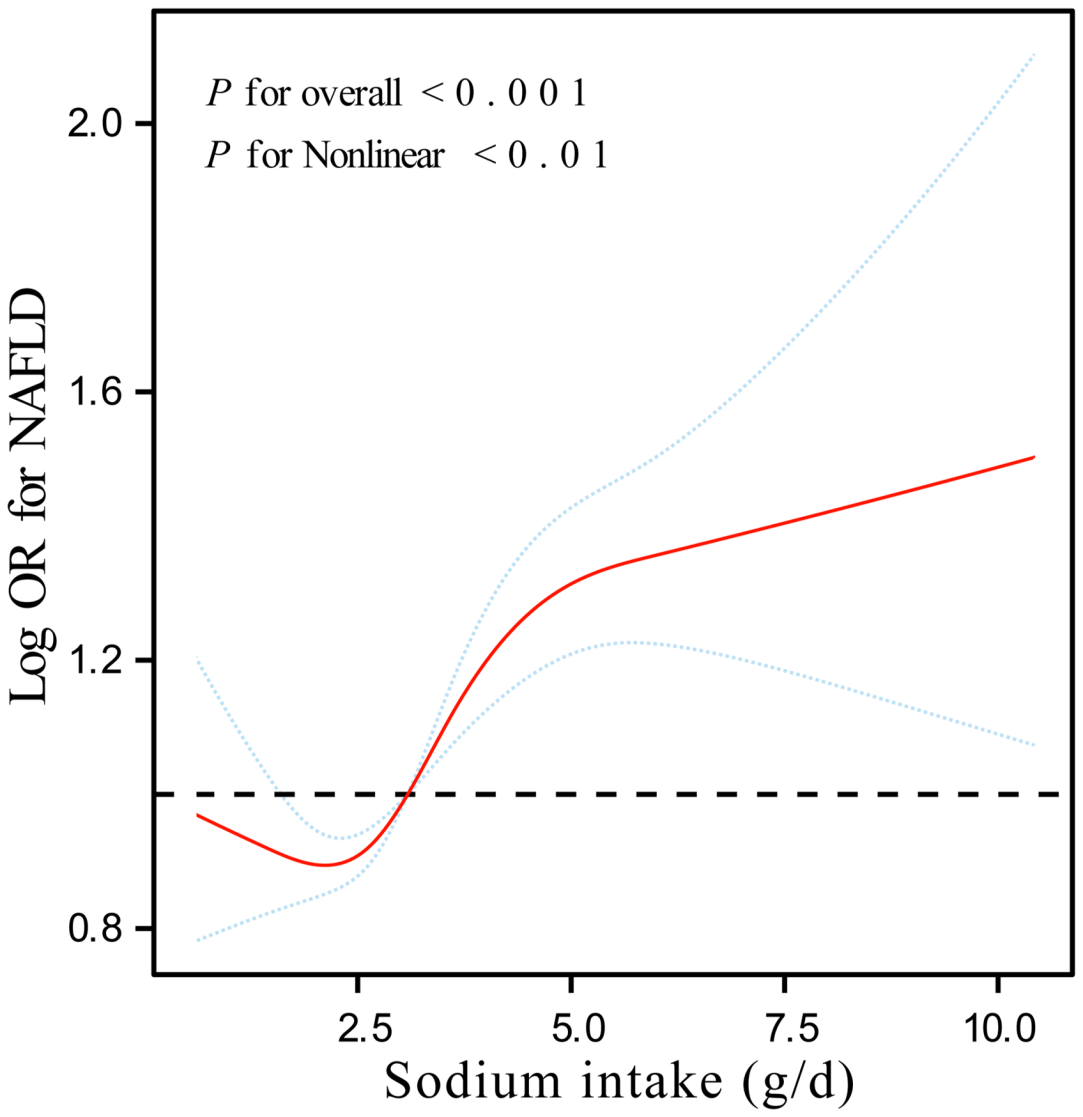
Smooth curve fitting illustrating the relationship between sodium intake and NAFLD prevalence, based on a generalized additive model.

**Figure 3:**
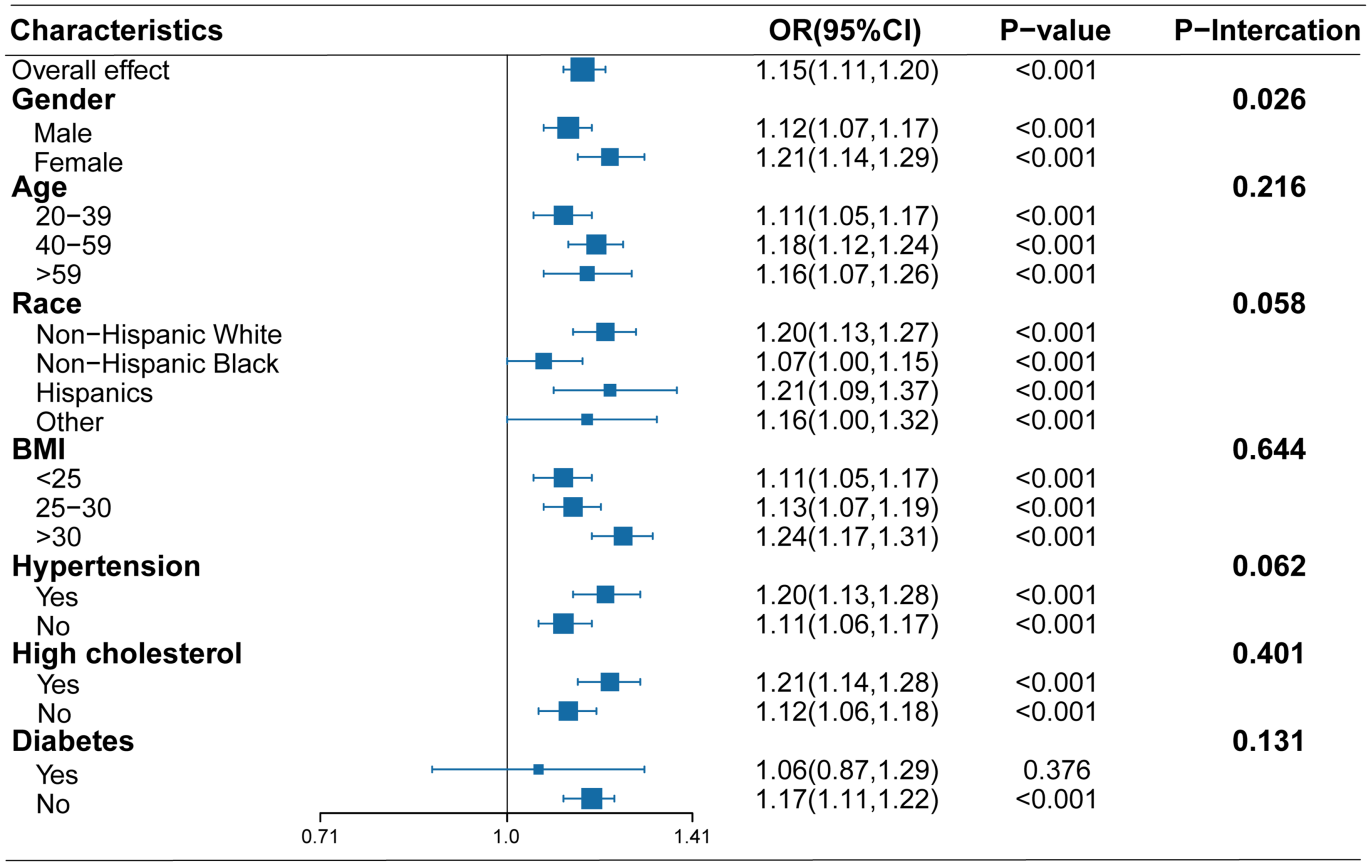
Subgroup analysis of the association between sodium intake and NAFLD Incidence, adjusted for confounders.

Smooth curve fitting (Figure 2) revealed a complex, non-linear association between sodium intake and NAFLD risk (overall *p* < 0.001, non-linear p < 0.001). We observed a decrease in NAFLD risk with increasing sodium intake at lower levels, but this trend reversed, with risk increasing after a certain threshold was surpassed. To further investigate this non-linear relationship, we performed a threshold effect analysis. The results in Table 3, using a two-piecewise linear model, identified an inflection point at 2.49 g/d. Specifically, when sodium intake was below 2.49 g/d, there was no significant association between sodium intake and NAFLD risk (OR = 0.94, 95% CI: 0.85–1.04, *p* = 0.226). However, when sodium intake exceeded 2.49 g/d, NAFLD risk was significantly and positively correlated with sodium intake (OR = 1.08, 95% CI: 1.05–1.12, *p* < 0.001). The likelihood ratio test (*p* = 0.020) supported the two-piecewise model, indicating that it more accurately describes the relationship between sodium intake and NAFLD risk compared to a single linear model. Additionally, we conducted multivariable-adjusted subgroup analyses based on variables such as age, gender, race/ethnicity, BMI, hypertension, high cholesterol, and diabetes (Figure 3). The findings indicated that the positive association between sodium intake and the prevalence of NAFLD was significantly stronger in females compared to males (male OR: 1.12, 95% CI: 1.07, 1.17; female OR: 1.21, 95% CI: 1.14, 1.29) (P-interaction = 0.026).

**Table 3:** Threshold effect analysis of the relationship of sodium intake with NAFLD.

	Adjusted OR (95% CI), *p*-value^a^
Fitting by the standard linear model	1.06 (1.03, 1.08) < 0.001
Fitting by the two-piecewise linear model	
Inflection point	2.49 g/d
Sodium intake < 2.49 g/d	0.94 (0.85, 1.04) 0.226
Sodium intake ≥ 2.49 g/d	1.08 (1.05, 1.12) < 0.001
P for Log-likelihood ratio	0.020

a Adjusted for sex, age, race, education, PIR, BMI, physical activity, smoking, total caloric intake, hypertension, High cholesterol, diabetes, ALT, AST, TC, TG, LDL, HDL, GGT.

### Association between sodium intake and mortality

As of December 31, 2019, the median follow-up period for this study was 6.7 years, with a quartile range of 3.8 to 9.8 years. During this period, a total of 2,301 participants (9.14%) died. Our analysis revealed a significant negative association between sodium intake and all-cause mortality in individuals with NAFLD. After comprehensive adjustment for multiple confounding variables in the weighted Cox regression analysis (Model 3), the HRs and 95% CIs for all-cause mortality across sodium intake quartiles (Q1-Q4) were as follows: 1.00 (reference group), 0.78 (0.67, 0.91), 0.68 (0.57, 0.81), and 0.63 (0.51, 0.77), with a trend test *p*-value of <0.001 (Table 4). When sodium intake was considered as a continuous variable, each additional unit of sodium intake was associated with a 12% reduction in all-cause mortality (HR: 0.88, 95% CI: 0.83, 0.93). This association was similarly observed in NAFLD participants, with a 13% reduction (HR: 0.87, 95% CI: 0.80, 0.96), and in non-NAFLD participants, with a 12% reduction (HR: 0.88, 95% CI: 0.81, 0.95).

**Table 4:** HR (95% CIs) for all-cause mortality based on sodium intake, weighted.

Sodium intake	Model 1			Model 2			Model 3		
	HR	95% CI	*p*-value	HR	95% CI	*p*-value	HR	95% CI	*p*-value
All participants									
Continuous^a^	0.76	0.73, 0.80	<0.001	0.88	0.82, 0.93	<0.001	0.88	0.83, 0.93	<0.001
Categorical^b^									
Q1	1.00	–	–	1.00	–	–	1.00	–	–
Q2	0.65	0.56, 0.76	<0.001	0.77	0.66, 0.90	<0.001	0.78	0.67, 0.91	0.002
Q3	0.50	0.43, 0.59	<0.001	0.67	0.56, 0.80	<0.001	0.68	0.57, 0.81	<0.001
Q4	0.39	0.33, 0.46	<0.001	0.62	0.50, 0.76	<0.001	0.63	0.51, 0.77	<0.001
P for trend			<0.001			<0.001			<0.001
NAFLD									
Continuous^a^	0.77	0.72, 0.83	<0.001	0.86	0.79, 0.95	0.002	0.87	0.80, 0.96	0.004
Categorical^b^									
Q1	1.00	–	–	1.00	–	–	1.00	–	–
Q2	0.74	0.58, 0.94	0.013	0.77	0.59, 1.01	0.056	0.79	0.61, 1.02	0.072
Q3	0.54	0.42, 0.70	<0.001	0.63	0.47, 0.85	0.003	0.66	0.49, 0.90	0.008
Q4	0.42	0.32, 0.55	<0.001	0.60	0.43, 0.84	0.003	0.63	0.45, 0.89	0.008
P for trend			<0.001			<0.001			<0.001
Non-NAFLD									
Continuous^a^	0.73	0.68, 0.79	<0.001	0.87	0.81, 0.95	0.001	0.88	0.81, 0.95	<0.001
Categorical^b^									
Q1	1.00	–	–	1.00	–	–	1.00	–	–
Q2	0.62	0.51, 0.76	<0.001	0.77	0.63, 0.96	0.018	0.79	0.64, 0.97	0.023
Q3	0.47	0.39, 0.57	<0.001	0.69	0.56, 0.85	<0.001	0.69	0.56, 0.85	<0.001
Q4	0.33	0.26, 0.43	<0.001	0.61	0.47, 0.79	<0.001	0.62	0.48, 0.81	<0.001
P for trend			<0.001			<0.001			<0.001

HR, hazard ratio, CI, confidence interval. Model 1: Non-adjusted. Model 2: Adjusted for sex, age, race, education, PIR. Model 3: Adjusted for sex, age, race, education, PIR, BMI, physical activity, smoking, total caloric intake, hypertension, High cholesterol, diabetes, ALT, AST, TC, TG, LDL, HDL, GGT.

a Each 1 unit increase in Sodium intake.

b Q1: <2.3 g/d; Q2: 2.3–3.1 g/d; Q3: 3.1–4.0 g/d; Q4: ≥4.0 g/d.

**Figure 4:**
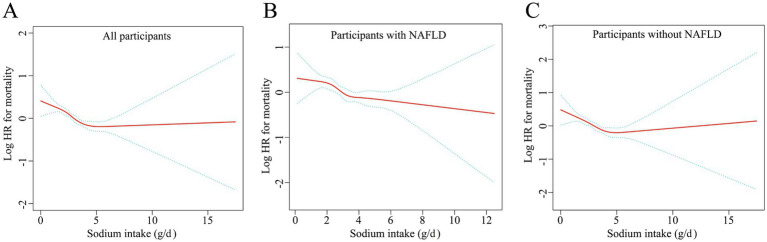
Smooth curve fitting of the relationship between sodium intake and all-cause mortality, separately in all participants **(A)**, participants with NAFLD **(B)**, and those without NAFLD **(C)**. Adjusted for sex, age, race, education, PIR, BMI, physical activity, smoking, total energy intake, hypertension, diabetes, hyperlipidemia, ALT, AST, TC, TG, LDL, HDL and GGT.

**Figure 5:**
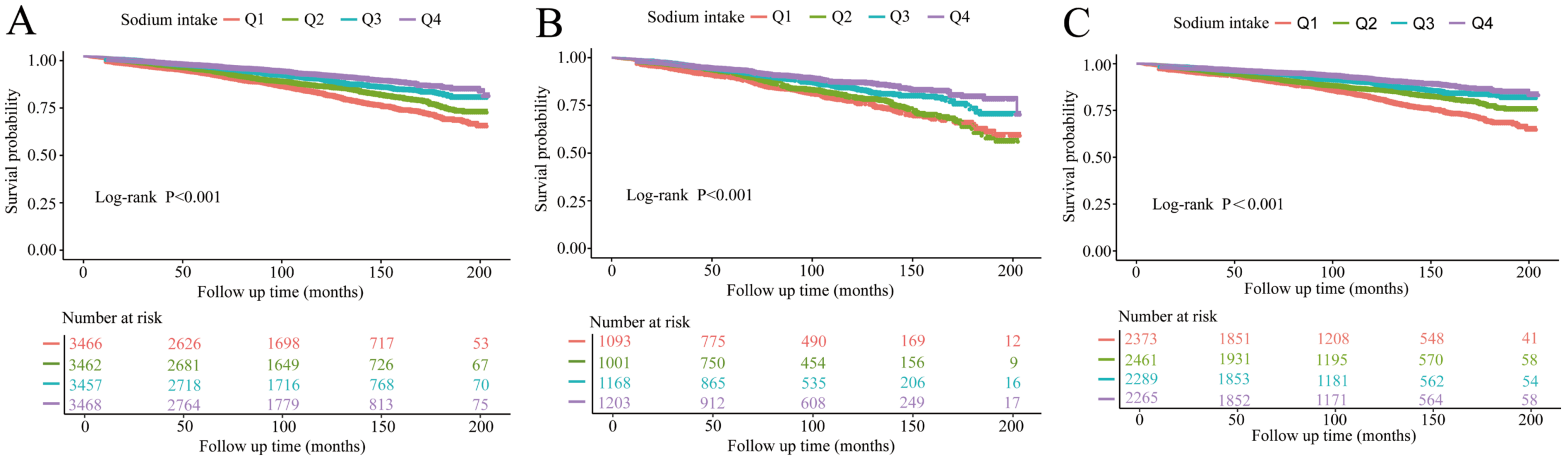
Kaplan–Meier analysis of all-cause mortality by quartiles of sodium intake (Q1-Q4) in **(A)** all participants, **(B)** participants with NAFLD, and **(C)** participants without NAFLD.

Smooth curve fitting further confirmed this negative association (all smooth curve fitting *p*-values <0.001, Figures 4A–C). Additionally, Kaplan–Meier survival curves demonstrated a negative correlation between sodium intake and all-cause mortality in the study population, regardless of NAFLD status (all log-rank *p*-values <0.001, Figures 5A–C).

In threshold analysis, participants with sodium intake below 3.5 g/day had a mortality risk ratio of 0.84 (95% CI: 0.80, 0.90, *p* < 0.001) (Table 5). This finding indicates that for every additional gram of sodium intake per day, the risk of death decreases by 16.0%. In populations consuming 3.5 grams or more of sodium daily, No meaningful correlation was observed between dietary sodium intake and mortality. This result suggests that for those exceeding a daily sodium intake of 3.5 grams, the risk of death does not further decrease with increased sodium consumption.

**Table 5:** Threshold effect analysis of the relationship of sodium intake with all-cause mortality.

	Adjusted HR (95% CI),*p*-value^a^
Fitting by the standard linear model	0.89 (0.86, 0.93) < 0.001
Fitting by the two-piecewise linear model	
Inflection point	3.5 g/d
Sodium intake < 3.5 g/d	0.84 (0.80, 0.90) < 0.001
Sodium intake ≥ 3.5 g/d	0.99 (0.90, 1.08) 0.784
P for Log-likelihood ratio	0.016

a Adjusted for sex, age, race, education, PIR, BMI, physical activity, smoking, total caloric intake, hypertension, High cholesterol, diabetes, ALT, AST, TC, TG, LDL, HDL, GGT.

## Discussion

As the first study to investigate the long-term survival impact of sodium intake on individuals with NAFLD, we found a positive correlation between daily sodium intake and NAFLD in this nationally representative U.S. population study. Specifically, this study revealed a non-linear relationship between sodium intake and the risk of NAFLD, with an inflection point at 2.49 grams per day. Beyond this inflection point, the risk of NAFLD increased significantly. Additionally, we were the first to investigate the association between sodium intake and all-cause mortality in patients with NAFLD. Surprisingly, we found a negative correlation when sodium intake was below 3.5 grams per day.

In the subgroup analysis for diabetes, the *p*-value for the diabetic group was not statistically significant. We attribute this gender difference to the loss of estrogen’s protective effects in postmenopausal women, which may increase the risk of fatty liver (23, 24). Future longitudinal studies are necessary to validate whether the relationship between sodium intake levels and NAFLD risk differs between male and female patients. In the subgroup analysis patients, the association between sodium intake and NAFLD was found to be non-significant. Possible reasons for this include: (1) Insufficient Sample Size: The sample size of diabetic participants may be too small to detect a significant association, particularly in subgroup analyses. (2) Confounding Factors: Diabetic participants may have other metabolic abnormalities (such as insulin resistance and hyperglycemia) that could obscure the relationship between sodium intake and NAFLD. (3) Physiological Mechanisms: Diabetes may influence the metabolism or function of sodium, altering its relationship with hepatic fat metabolism.

Sodium primarily occurs as sodium chloride and is a prevalent element in contemporary diets, largely due to the extensive intake of processed and fast foods (25). Sodium intake is associated with increased risks of obesity (26), insulin resistance (27), type 2 diabetes (13, 28), and metabolic disorders (29). Given that these metabolic disorders are linked to NAFLD (30, 31), the impact of sodium intake on the risk of developing and progressing NAFLD has received growing attention. Prior research has indicated that elevated sodium consumption is independently linked to a higher risk of NAFLD and liver fibrosis (10, 32). However, one study found no correlation between sodium intake and the risk of NAFLD in older individuals (33), while a recent study suggested that high sodium intake may be a significant factor leading to NAFLD in women, but not in men (34).

Our research, which included 13,853 participants from 2003 to 2018, demonstrated a positive correlation between high sodium intake and the occurrence of NAFLD through logistic regression analysis, corroborating most previous studies. There are several potential mechanisms linking high salt intake and NAFLD.

The primary pathological mechanisms of NAFLD include excessive fat accumulation and insulin resistance (35). Research indicates that high salt intake can induce insulin resistance (36) and promote the increase of white adipose tissue (37), contributing to the development of NAFLD. Animal studies have shown that a high-salt diet may reduce the liver’s antioxidant capacity and promote liver inflammation and fibrosis (38). Furthermore, high salt intake leads to increased sodium content in liver tissue and decreased mitochondrial respiratory function, with mitochondrial dysfunction being associated with hepatocyte steatosis (39). Leptin plays a protective role in preventing ectopic fat accumulation and lipotoxicity, and leptin resistance is considered a significant factor in the progression of fatty liver (40). High salt intake may also activate the aldose reductase-fructokinase pathway, leading to increased endogenous fructose production, which in turn induces leptin resistance (41).

Hypertension has also been demonstrated to contribute to the development of NAFLD (42). Given that numerous studies indicate a direct correlation between sodium intake and blood pressure, the effect of sodium on blood pressure regulation is regarded as a potential mechanism contributing to NAFLD. High sodium dietary intake can induce changes in the extracellular matrix of arterial wall cells, leading to arteriosclerosis (43), which alters hepatic hemodynamics, increases the burden on the liver, and promotes fat accumulation in the liver.

In the Cox regression analysis, our findings appear to contradict conventional wisdom. We discovered that increased sodium intake was associated with reduced mortality among patients with NAFLD. Threshold effect analysis demonstrated that when sodium intake does not exceed 3.5 grams per day (equivalent to 8.9 grams of sodium chloride), all-cause mortality among NAFLD patients decreases as sodium intake increases. For decades, practicing physicians have warned their patients against excessive salt consumption. Entities such as the World Health Organization (WHO) have released guidelines recommending a reduction in habitual salt intake, typically to less than 2 grams of sodium (equivalent to 5 grams of salt) (44), not only for those with hypertension but also for individuals with normal blood pressure.

In 2020, a global study involving 181 countries published in the authoritative cardiovascular journal European Heart Journal reached a contrary conclusion, suggesting that sodium intake is positively correlated with life expectancy and negatively correlated with all-cause mortality in both global and high-income countries (45), which appears to align with our findings. A 2018 study published in The Lancet demonstrated that sodium intake elevates the risk of cardiovascular disease and stroke, but this was only significant in regions where the average sodium intake exceeded 5 grams per day; this relationship did not apply to other communities and countries (46).

Moreover, an increasing number of prospective studies suggest a J-shaped relationship between sodium intake and cardiovascular events (47–49). Moderate sodium intake appears to have physiological benefits for cardiovascular health, while both high and low intake levels may have pathological effects. This reflects a predicted relationship between an essential nutrient and health outcomes. Therefore, sodium intake should be viewed as having a U-shaped relationship with health; both excessive and insufficient intake may increase the risk of mortality. Thus, advocating for a blanket reduction in sodium intake without considering individual circumstances may be unreasonable.

Our study has several limitations. First, we used the US Fatty Liver Index (US-FLI) for the diagnosis of NAFLD rather than liver biopsy, which may limit the accuracy of the diagnosis. Additionally, dietary data were based on self-reports instead of the Definitive standard for sodium intake assessment, which is 24-h urinary sodium excretion. Self-reported dietary information may be susceptible to recall bias or reporting bias, impacting the accuracy of sodium intake measurements. Furthermore, our inability to systematically evaluate secondary etiologies—particularly autoimmune hepatitis and steatogenic medications—prevents definitive exclusion of secondary hepatic steatosis.

## Conclusion

In conclusion, this study indicates that increased sodium intake in the diet of NAFLD patients is linked to the occurrence of NAFLD and beneficially related to a lowered risk of all-cause mortality. The dose–response relationship exhibits a nonlinear pattern, with a critical inflection point occurring at an intake of approximately 3.5 grams per day. These findings emphasize the need for establishing dietary sodium intake standards, particularly for individuals with NAFLD. Further investigations are necessary to validate these findings and to gain a deeper understanding of the underlying processes linking sodium intake to the pathogenesis and all-cause mortality among individuals with NAFLD.

## Data Availability

Publicly available datasets were analyzed in this study. This data can be found at: https://www.cdc.gov/nchs/nhanes/index.htm.
