# Supplementary material for: The relationship between dietary sodium intake and all-cause mortality in patients with non-alcoholic fatty liver disease: a cohort study from NHANES 2003–2018
Source: Front Nutr. 2025 Apr 23;12:1530025. doi: 10.3389/fnut.2025.1530025 (PMC12055778; doi:10.3389/fnut.2025.1530025)
Supplement: Supplementary file 1 [file Table_1.docx]

**Table S1** Summary of characteristics and results from human studies.

| **Authors (Year of Publication)** | **Design** | **Country of origin** | **Population** | **Year of collection** | **Participants (total)** | **Sodium intake (method)** | **NAFLD diagnosis** | **Results** |
| --- | --- | --- | --- | --- | --- | --- | --- | --- |
| Jihye Lee et al. (2024) | prospective cohort design | Korean | Healthy adults | 2001 - 2002 | 2582 | 24-h urinary sodium excretion levels | hepatic steatosis index | Risk factor in females, not in males |
| Takahashi et al. (2022) | Cross-sectional | Japan | Type 2 Diabetes | 2016–2018 | 310 | Tanaka’s formula | Predictive formulas | >9.5 g/day vs. <9.5  g/day sodium = 1.76  (1.02–3.03) |
| Luo et al. (2022) | Cross-sectional | China | Adults (18–59 yo) | 2017–2019 | 23,867 | Tanaka’s formula | Ultrasound | Q4 vs. Q1 = 1.60  (1.47–1.76) |
| Zhou et al. (2021) | Cross-sectional | USA | Noninstitutionalized  adults (>20 yo) | 2007–2017 | 11,022 | 24-h food recall (2  evaluations) | Predictive formulas | with BMI (without  HAS)–HIS: Q4 vs.  Q1 = 1.30 (1.04; 1.64) |
| Emamat et al. (2021) | Cross-sectional | Iran | People with NAFLD  and controls with  pancreaticobiliary  disorders | 2015 | 999 | FFQ | FibroScan | T3 vs. T1, = 2.42  (1.13–5.15 |
| van den Berg et al. (2019) | Cross-sectional | Netherlands | Adults with macro  albuminuria and  controls | 2001-2003 | 6132 | 24-h uNa+ | Predictive formulas | For each SD of  sodium (55.99  mmol/L)–HSI = 1.40  (1.31–1.51); FLI =  1.30 (1.21; 1.41) |
| Choi et al. (2016) | Cross-sectional | South Korea | Healthy adults | 2011–2013 | 100177 | FFQ | Ultrasound | Q5 vs. Q1 (with  BMI): Male: 1.16  (1.10, 1.22); Female:  1.11 (0.99, 1.24) |
| Huh et al. (2015) | Cross-sectional | South Korea | Noninstitutionalized  adults (>25 yo) | 2010–2013 | 27,433 | Tanaka’s formula | Predictive formulas | T3 vs. T1-HSI = 1.39  (1.26–1.55); FLI: =  1.29 (1.39–2.20).  For each SD-HSI =  1.21 (1.16; 1.26); FLI  = 1.29 (1.19; 1.41) |
| Portela et al. (2015) | Cross-sectional | Brazil | hypertensive elder patients | 2009.6-2009.12 | 229 | Three 24-h dietary  recalls | hepatic steatosis index | Not associated |

BMI = body mass index; HSI = hepatic steatosis index; FLI = fatty liver index; M = male; F = female; CAP = controlled attenuation parameter; OR = odds ratio; PR = prevalence ratio; CI = confidence interval; SBP = systolic blood pressure;SD = standard deviation. Ultrasound-assessed NAFLD cutoff = determined by the presence of a diffuse increase infine echoes in the liver parenchyma compared to the kidney or spleen parenchyma.
